# Understanding the Effect of Particle Size and Processing on Almond Lipid Bioaccessibility through Microstructural Analysis: From Mastication to Faecal Collection

**DOI:** 10.3390/nu10020213

**Published:** 2018-02-14

**Authors:** Giuseppina Mandalari, Mary L. Parker, Myriam M.-L. Grundy, Terri Grassby, Antonella Smeriglio, Carlo Bisignano, Roberto Raciti, Domenico Trombetta, David J. Baer, Peter J. Wilde

**Affiliations:** 1 Department of Chemical, Biological, Pharmaceutical and Environmental Science, University of Messina, Viale SS. Annunziata, 98168 Messina, Italy; gmandalari@unime.it (G.M.); asmeriglio@unime.it (A.S.); rraciti@unime.it (R.R.); dtrombetta@unime.it (D.T.); 2 Quadram Institute Bioscience, Norwich NR4 7UA, UK; mary.parker@qibextra.co.uk (M.L.P.); myriam.grundy@quadram.ac.uk (M.M.-L.G.); 3 School of Biosciences and Medicine, Faculty of Health and Medical Sciences, University of Surrey, Guildford GU2 7XH, UK; t.grassby@surrey.ac.uk; 4 Department of Biomedical, Dental, Morphological and Functional Images Sciences, University of Messina, Via C. Valeria, 98125 Messina, Italy; cbisignano@unime.it; 5 U.S. Department of Agriculture, Agricultural Research Service, Beltsville Human Nutrition Research Centre, Building 307B, Room 213, BARC-East, Beltsville, MD 20705, USA; David.Baer@ARS.USDA.GOV

**Keywords:** almonds, particle size, lipid bioaccessibility, microstructural analysis

## Abstract

We have previously reported on the low lipid bioaccessibility from almond seeds during digestion in the upper gastrointestinal tract (GIT). In the present study, we quantified the lipid released during artificial mastication from four almond meals: natural raw almonds (NA), roasted almonds (RA), roasted diced almonds (DA) and almond butter from roasted almonds (AB). Lipid release after mastication (8.9% from NA, 11.8% from RA, 12.4% from DA and 6.2% from AB) was used to validate our theoretical mathematical model of lipid bioaccessibility. The total lipid potentially available for digestion in AB was 94.0%, which included the freely available lipid resulting from the initial sample processing and the further small amount of lipid released from the intact almond particles during mastication. Particle size distributions measured after mastication in NA, RA and DA showed most of the particles had a size of 1000 µm and above, whereas AB bolus mainly contained small particles (<850 µm). Microstructural analysis of faecal samples from volunteers consuming NA, RA, DA and AB confirmed that some lipid in NA, RA and DA remained encapsulated within the plant tissue throughout digestion, whereas almost complete digestion was observed in the AB sample. We conclude that the structure and particle size of the almond meals are the main factors in regulating lipid bioaccessibility in the gut.

## 1. Introduction

The behaviour of almonds in the gastrointestinal tract (GIT) may explain why almonds have potential health benefits and reduce risk factors associated with type 2 diabetes, cardiovascular disease, cancer and obesity [1,2,3]. Previous studies have established that almond cell walls play a crucial role in regulating nutrient bioaccessibility in the GIT [4,5]. The term ‘bioaccessibility’ is defined as the proportion of a nutrient or phytochemical compound ‘released’ from a complex food matrix during digestion and, therefore, potentially available for absorption in the GIT. Using an in vitro and an in vivo study, we have recently demonstrated that test meals containing almonds of different particle sizes behaved differently: the degree of lipid encapsulation affected the rate and extent of bioaccessibility in the upper GIT [6]. We have also demonstrated that mastication of natural raw almonds released only a small proportion (7.9%) of the total lipid and was only slightly higher for roasted almonds (11.1%) [7]. The lipid release from masticated almonds was in close agreement with that predicted by a theoretical model for almond lipid bioaccessibility [7,8]. Using an in vitro model of duodenal digestion [9], it was observed that a decrease in almond particle size resulted in an increased rate and extent of lipolysis.

Novotny et al. [10] conducted a feeding study in healthy adults to determine the energy value of almonds as a representative food from a group for which the Atwater factors may overestimate the energy value. They showed that only 76% of the energy contained within almonds (based on the Atwater factors) was actually metabolised [10]. Furthermore, when calculating the metabolisable energy (ME) of whole natural almonds, whole roasted almonds, chopped almonds and almond butter, it was demonstrated that the number of calories absorbed was dependent on the form in which almonds were consumed.

Based on these findings, the aims of the present work were: (a) to investigate the mechanisms responsible for the loss in observed in vivo metabolisable energy compared to that calculated from nutrient composition using the Atwater general factors; this was performed by microscopy in post-GIT faecal samples; (b) to carry out microstructural investigations on freshly artificially “masticated” almond samples, to determine the particle size distribution of the “masticated” samples and the extent of lipid release after oral digestion; and (c) to further validate the mathematical model previously developed to predict lipid release from masticated almonds [8].

## 2. Materials and Methods

### 2.1. Almond and Faecal Samples

Four almond types all with brown testa present (natural raw almonds, NA, roasted almonds, RA, roasted diced almonds, DA and almond butter, AB) were provided by the Almond Board of California. Smooth unsalted AB was industrially produced by grinding unskinned roasted almonds. It contained (as per label): fat (50%, of which 4.7% were saturated), total carbohydrates (25%, of which 12.5% were dietary fibre and 6.2% were sugars), protein (15.6%). Faecal samples were collected from humans who were participants in a study to measure the metabolisable energy of the almonds [11]. This feeding study was a crossover, randomized control trial. Volunteers (10 men and 8 women) were fed the 5 distinct feeding regimes (control, NA, RA, DA and AB) as part of a highly controlled diet. During the feeding periods, all meals (using a 7-day menu cycle) for the volunteers were prepared at the Beltsville Human Nutrition Research Centre (the Centre) and Monday through Friday breakfast and dinner were consumed at the Centre under supervision of the research investigators. Lunch and weekend meals were prepared at the Centre and packaged for consumption off-site. Foods for all meals and snacks were identical (except for the form of nut). Food for all meals was prepared by weight, to the nearest 1 g, to produce daily menus providing a range in energy from 1600 kcal to 4000 kcal. Volunteers were fed the energy needed to maintain their body weight (body weight was measured each morning, Monday through Friday) and adjustments to the amount of food consumed was made by increasing or decreasing the amount of all food, proportionately, such that the composition of the diet was identical for all volunteers, independent of the energy they required to maintain body weight. A total of 42 g/day of each form of nut (NA, RA, DA and AB) was consumed daily with half the amount consumed at breakfast and the other half consumed at dinner. For the control diet, the amount of all foods was increased proportionately such that the energy content of the control diet was designed to be equal to the 4 four diets that contained the control foods plus the nuts. Volunteers were recruited from the area around the Centre and were screened to insure they met the study criteria. Briefly, subjects were healthy individuals (not taking any medications or supplements that might interfere with study outcomes) without dental or digestive conditions. At the beginning of the study, the mean (±SEM (Standard error of the mean)) age of the volunteers was 56.7 ± 2.4 year, their mean height was 170.2 ± 2.1 cm and mean weight 88.6 ± 5.6 kg.

From each of the 18 volunteers, faecal samples were collected from the beginning and end of each of 5 distinct feeding regimes (control, NA, RA, DA and AB) (each treatment period lasting 3 weeks). Following a 14-day adaptation to each of the 5 feeding regimes, the study subjects received blue dye capsules to mark the beginning of a one week excreta collection period and a second blue dye capsule to mark the end of the collection period.

The study protocol and informed consent form were reviewed and approved by the MedStar Health Research Institute and the associated faecal samples were registered with the QIB Human Research Governance Committee in December 2016.

### 2.2. Simulated Oral Digestion

The aim of this procedure was to simulate the chewing of the almond meals in the mouth. Chewing is the initial step in the digestion process and this procedure was designed to simulate both the salivary amylase activity and the mechanical breakdown of the food. Four almond samples, NA, RA, DA and AB (25 g), were minced 3 times using a mincer (Lexen mincer, Windermere, UK) to simulate the mechanical oral breakdown of the meal. Thereafter, 12.5 mL of Simulated Salivary Fluid (SSF) at pH 6.9 (0.15 M sodium chloride, 3 mM urea) and 900 U Human Salivary Amylase (HSA) dissolved in 1 mL SSF were added to the minced almonds or the almond butter [12] and mixed. This process produced a paste of equal ratio of solid to water as calculated from human chewing [13].

### 2.3. Particle Size Distribution (PSD)

The particle size of the samples before (AB) and after simulated oral digestion (NA, RA, DA and AB) was measured using mechanical sieving. Briefly, 22.5 g of each sample mixed with SSF was loaded on a stack of sieves with 9 aperture sizes: 3350, 2000, 1000, 850, 500, 250, 125, 63 and 32 µm (Endecott test sieve shaker, Endecotts Ltd., London, UK). The samples were washed with deionized water, shaken for 15 min and washed again, thus ensuring separation of the particles. The sieves were then dried in a forced-air oven at 56 °C for 6 h. The bases were left to dry at 100 °C overnight (about 15 h), which permitted complete evaporation of the water. The sieves were weighed before loading the sample and then again after having been oven dried. The dried fractions retained on each sieve and the base were expressed as a percentage of the weight of almonds before simulated oral processing.

### 2.4. Lipid Release after Oral Processing and Mathematical Model

Lipid extraction for total fat determination on all samples, before and after oral processing, was performed with a Soxhlet automatic Soxtec 2050 extraction (FOSS Analytical, Hilleroed, Denmark) using *n*-hexane as a solvent [5]. For AB, it was assumed that the continuous oil phase was bioaccessible and therefore the aim was to measure the additional lipid released from the almond particles present in AB. The almond particles were separated from the continuous oil phase of AB by centrifugation (REMI Elektrotechnik LTD., Vasai (East), India (13,000× *g*, 15 min) before and after chewing and the lipid content of the particles was determined as described below. The fat present in the pellets (almond particles) was also determined: the pellet was washed 5 times with warm (37 °C) distilled water to remove any free fat released from the cells, then separated by centrifugation and quantified by *n*-hexane extraction. The lipid present within the remaining wet pellet (theoretically inside the almond cells) was extracted by the Bligh & Dyer [14] method. Briefly, almond particles were extracted in chloroform/methanol/water in the proportions 1:2:0.8 and the lipid was then quantified in the chloroform layer.

### 2.5. Results of Lipid Content Were Expressed as a Percentage of Dry Weight

Lipid release was predicted from the measured particle size distribution by sieving and the previously measured average cell diameter, 36 µm using the method of Grassby et al. [8]. The threshold diameter below which 100% release would be achieved was 54 µm, particles below the threshold were not included in the calculations of lipid release. The spreadsheet provided as supplementary information was modified to accept particle size data from sieving alone and to account for the particles above the threshold diameter that were recovered on the 63 µm sieve (50 µm < *p* < 100 µm).

### 2.6. Microstructural Analysis

Portions of each almond sample (NA, RA, DA and AB), before and after simulated oral digestion, were incubated in the chelating agent CDTA (1,2-Cyclohexylenedinitrilotetraacetic acid) (50 mM CDTA, pH = 7) at 4 °C for a minimum of 4 weeks [15]. This treatment weakens the pectin layer in the middle lamella between cells so that individual cells can be separated from lumps of tissue by gentle pressure. These individual cells were then observed, unstained, by microscopy (bright-field or polarizing optics), or after staining with Sudan IV (0.1% Sudan IV in 1:1 acetone and 70% ethanol) to visualize their oil content. The CDTA treatment has previously been found to prevent microbial growth and to retain the oil fraction in the form it exists in the cells of the kernel, either as individual oil bodies in NA or as large coalesced oil globules in RA which are characteristic of the roasting process [15]. Fresh sections of raw and roasted samples were not used because the sectioning process was found to release the oil from the damaged cells so that the spatial information was lost.

Initial observations were made on faecal samples from 3 randomly-chosen volunteers on material stored in CDTA [15]. It was found that this method was not optimal as some of the free oil content rose to the surface and larger lumps of tissue sank in the CDTA making it difficult to obtain a representative sample. However, it was a useful preliminary step to help identify the range of plant structures that survived passage through the bowel. These included wheat bran layers (primarily aleurone, the cells of which are similar in size to almond tissue) and brush hairs, vascular tissue, xylem and tannin body inclusions.

Subsequent investigations on samples from volunteers with measured high faecal fat content were made by mixing a small sample of the frozen faecal matter directly on a microscope slide with the oil stain Sudan IV to minimise loss of components.

For microscopy, samples were examined and photographed using an Olympus BX60 (Olympus, Southend-on-Sea, UK) microscope and ProgRes^®^ Capture Pro 2.1 software (Jenoptik, Jena, Germany).

### 2.7. Statistical Analysis

Results of lipid release from mastication were expressed as mean ± standard deviation (SD) of four independent experiments and analysed by one-way analysis of variance (ANOVA). The significance was assayed by using the Student-Newman-Keuls test using the SigmaPlot 12.0 software (Systat Software Inc., San Jose, CA, USA). Statistical significance was considered at *p* < 0.001.

## 3. Results

### 3.1. Particle Size Analysis

**Figure 1:**
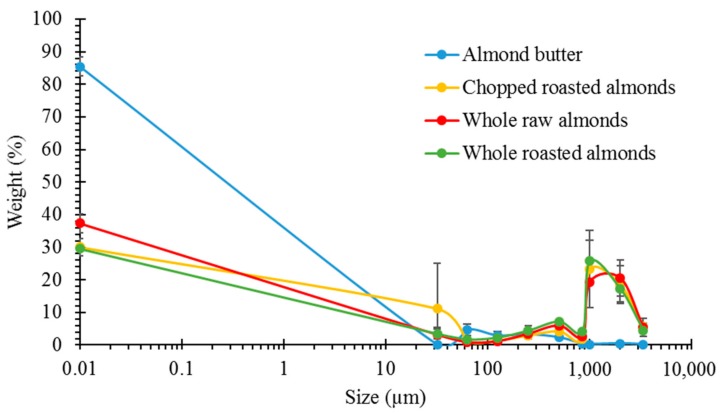
Particle size distributions by mechanical sieving of almond boluses (*n* = 2). The weight % of all material recovered in the sieve base (<32 µm) is given at size = 0.01 µm.

The weight of masticated almond retained on the sieves, presented as a percentage of the original weight of the masticated almond, was plotted against the aperture size of each sieve. The average PSDs for the different almond samples is shown in Figure 1. NA, RA and DA had very similar PSDs with most of the particles having a size of 1000 µm and above. On the other hand, AB bolus contained mainly small particles (<850 µm). The PSDs of NA and RA were similar to the ones measured for boluses from our human study [7], demonstrating that our simulated oral processing was a good alternative to human mastication.

### 3.2. Lipid Release after Simulated Mastication and Predicted Lipid Release

The release of total lipid as a percentage of the original lipid content of each sample (53.4%, *w*/*w* for NA, 54.1%, *w*/*w* for RA, 55.6%, *w*/*w* for DA and 50.1%, *w*/*w* for AB) after simulated mastication is reported in Table 1. In agreement with previous data [15], between 8.9% and 11.8% of the original lipid in the NA and RA samples, respectively, was released as a result of mastication. The higher lipid release in roasted DA compared with that detected in RA could be explained by the increased surface area in DA, which were roasted after dicing. For AB, to calculate the total available lipid, we had to determine the additional lipid released from almond particles in AB as a result of mastication. The lipid released from particles in AB following chewing (6.2%) was calculated as % lipid content of the remaining intact almond tissue after the free lipid in the continuous-oil phase (48.2% of total lipid) and the available lipid associated with the almond particles (39.6% of total lipid) had been removed (see Section 2.4). Therefore, the total lipid available for digestion in AB, obtained by combining the available lipid due to initial processing (continuous phase lipid plus available lipid associated with the particles) and the additional lipid released from the remaining intact particles during mastication, was 94.0%.

The values for predicted lipid release are presented in Table 1: a close agreement with the measured lipid release was obtained with NA, RA and AB. The measured lipid release was higher than the predicted release in DA: this is probably the effect of roasting.

### 3.3. Microscopy Examination on Almond Baseline Samples

Observations on baseline samples are essential to characterize the differences in microstructure between natural raw almond tissue, almond tissue processed by roasting and almond tissue roasted and ground to butter. These differences are relevant to the behaviour of the almond material during chewing and subsequently to the fate of the almond tissue in the digestive tract.

#### 3.3.1. NA

**Figure 2:**
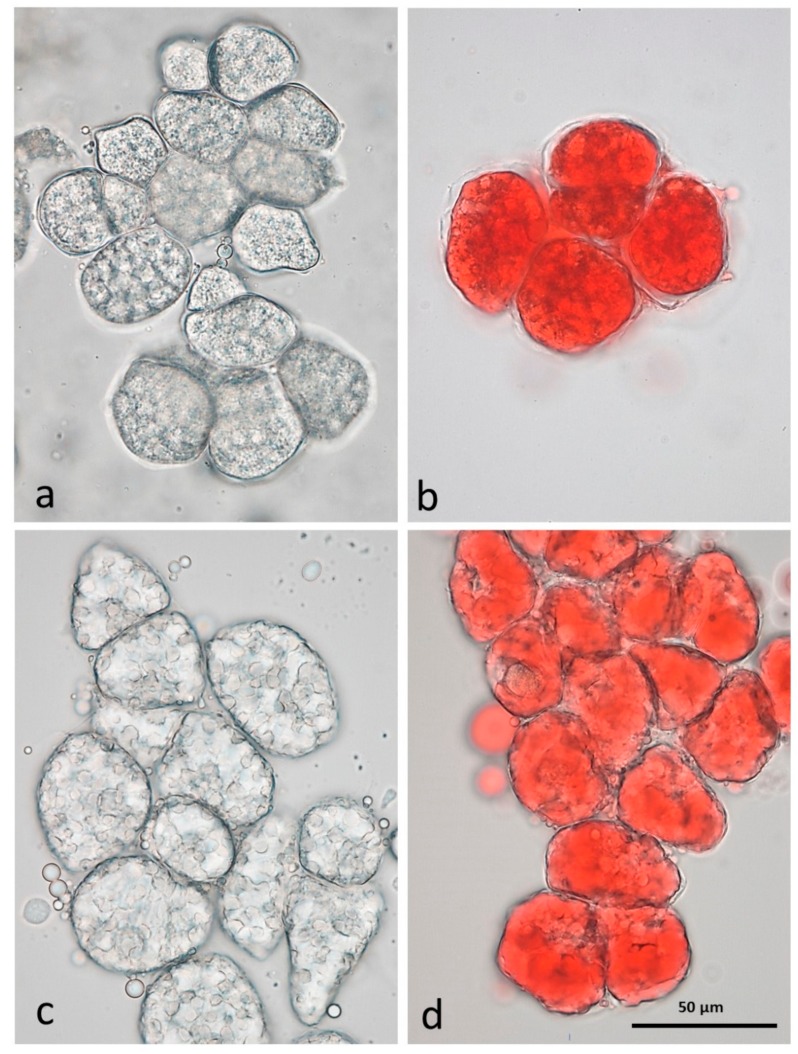
CDTA-separated cells of baseline natural raw almonds (**a**) unstained (**b**) lipid stained with Sudan IV and roasted almonds (**c**) unstained (**d**) lipid stained with Sudan IV showing lipid coalescence in the cells following roasting.

Individual whole cells separated from raw almond tissue by CDTA are shown unstained in Figure 2a and stained with Sudan IV to locate lipid (Figure 2b). The cells are small, less than 50 µm in diameter and tightly packed, with well-defined cell walls. The appearance and distribution of the protein bodies and lipid in unstained cells after CDTA treatment is consistent with previous observations [15] that the lipid is still within oleosomes, surrounding the protein bodies as in mature raw almonds not treated with CDTA. In Sudan IV-stained material, the survival of the oleosomes is demonstrated by an even distribution of lipid within each cell (Figure 2b).

#### 3.3.2. RA

The effect of roasting, in both whole and diced almonds, is to liberate the lipid from the oil bodies, which then forms large lipid droplets, as seen in both unstained (Figure 2c) and stained cells (Figure 2d). It is possible that individual cells in chopped almonds may achieve a higher internal temperature than those in whole roast almonds due to the greater surface area exposed to roasting.

#### 3.3.3. AB

**Figure 3:**
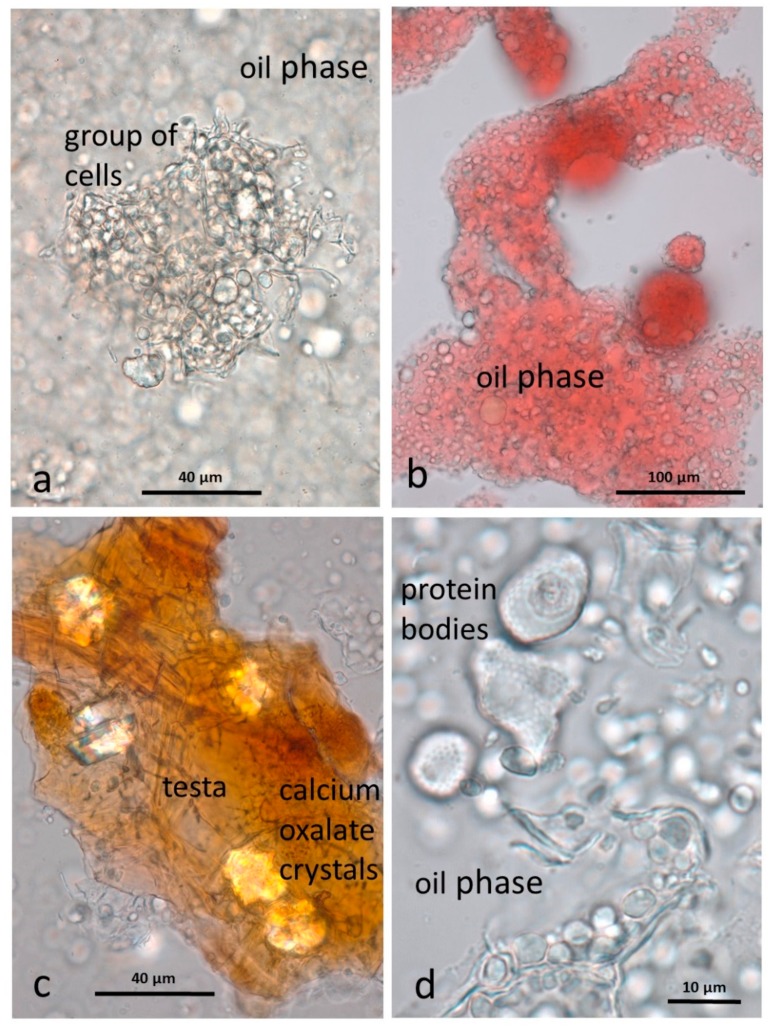
Almond butter baseline sample showing cellular fragments suspended in a lipid phase (**a**) a small group of kernel cells from which the lipid has been released (**b**) the lipid phase stained with Sudan IV (**c**) fragments of brown testa containing large calcium oxalate crystals (**d**) protein bodies and cell wall fragments in the lipid phase.

The baseline AB sample differed considerably from the raw and roasted baseline samples in that most of the cells of the kernels are ruptured, releasing the lipid (and other cell contents) to form a paste. Cell fragments (walls, protein bodies, nuclei, testa and some small intact clumps of cells) are suspended in a continuous lipid phase (Figure 3a), which was stained with Sudan IV (Figure 3b). Fragments of the brown testa are rich in calcium oxalate crystals (Figure 3c). Protein bodies, liberated from the cells by the grinding process, retain the dimpled surface impressions created by the surrounding oleosomes (Figure 3d). The other major particulate components of the almond butter are fragments of cell walls (Figure 3d).

**Figure 4:**
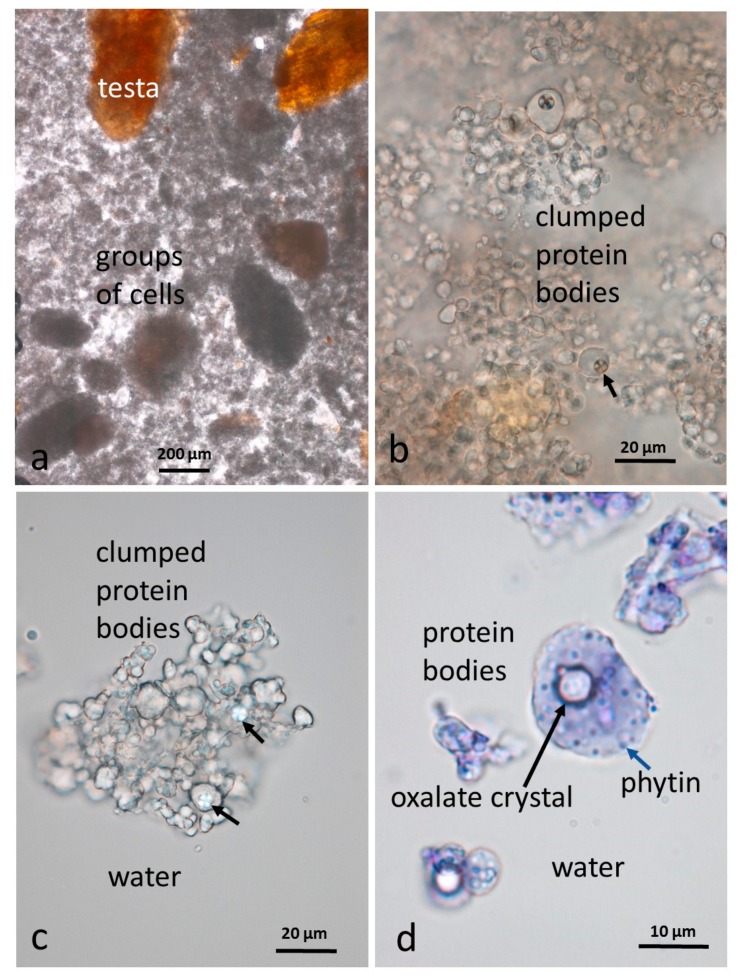
Almond butter baseline sample de-oiled in chloroform/methanol: (**a**) The butter contains clumps of kernel cells and testa fragments; (**b**,**c**) individual protein bodies form clumps with some containing small crystals of calcium oxalate (black arrows) visible under polarising optics; (**d**) protein bodies stained with dilute toluidine blue showing an oxalate crystal and phytin globoids (blue arrow) found in the larger protein bodies.

Additional information on the components of the almond butter starting material, particularly the protein bodies, was obtained by first de-oiling the butter with chloroform/methanol (1:1). The material could then be viewed more clearly (Figure 4) unstained, or stained with dilute aqueous toluidine blue. Although the butter looks finely ground, it does contain some multicellular particles in the range 150 µm–1 mm as illustrated in Figure 4a. Following de-oiling, the protein bodies tended to clump together but small crystals of calcium oxalate are visible within the larger protein bodies (Figure 4b–d, black arrows) under polarizing optics. Also visible within the larger protein bodies, after staining with dilute toluidine blue, are spherical globoid bodies in the form of small spheres (Figure 4d, blue arrow). These globoids have been shown by EDX (Energy Dispersive X-ray) analysis to be rich in phytin [16], the main site for mineral storage in seeds. It should be noted that the protein bodies and their contents, released from the cells by grinding, are far more accessible to digestive enzymes that those still within cell walls [17].

### 3.4. Microscopy Examination after Simulated Oral Digestion

#### 3.4.1. Chewed NA

**Figure 5:**
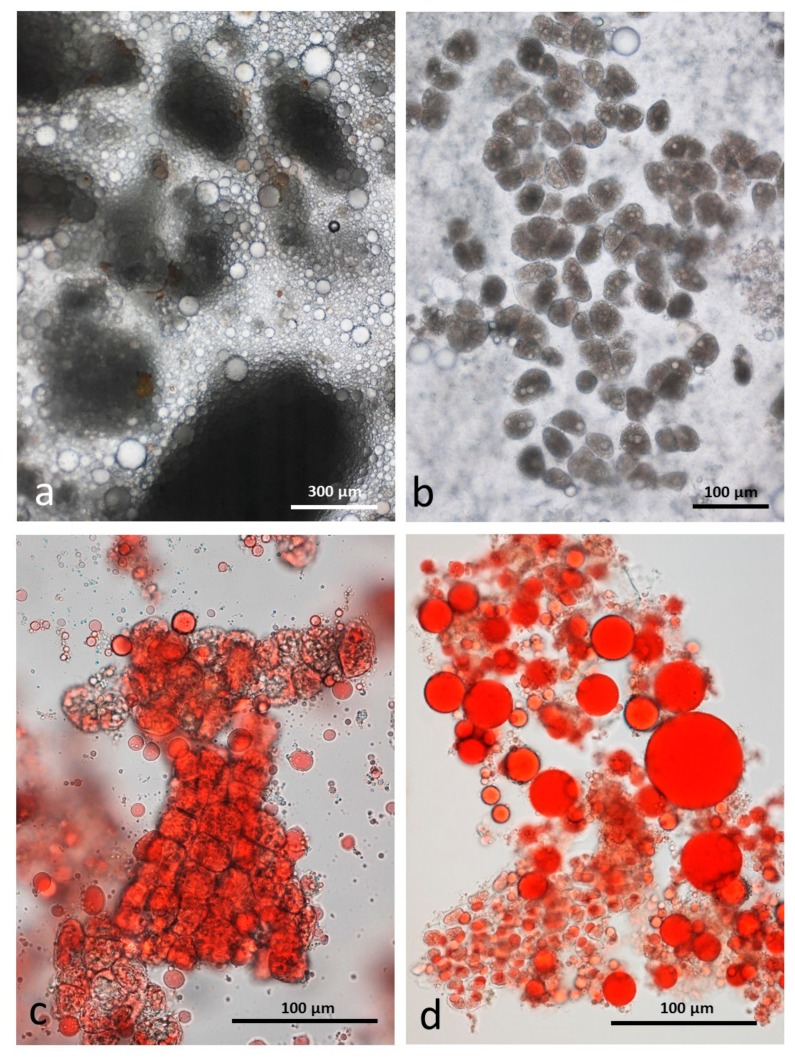
Chewed whole raw almonds stored in CDTA: (**a**) multicellular particles of almond tissue surrounded by released lipid drops; (**b**) individual undamaged cells separated from the larger particles contain lipid still within oleosomes; (**c**) Sudan IV staining showing the even distribution of lipid in oleosomes within whole cells; (**d**) lipid released from damaged cells coalesces into larger drops without inclusions.

The size range of multicellular particles of the unstained chewed raw sample at low magnification is shown in Figure 5a. Lipid which has been expressed by the chewing process is present as droplets of different sizes. In this sample, which has undergone artificial chewing and storage in CDTA, the lipid droplets appear to be a single lipid phase. However, preliminary observations on fresh volunteer-chewed NA showed that many of the larger lipid drops were in the form of water-in-oil-in-water (WOW) compound emulsions but the internal water droplets tended to coalesce over time. It is possible that storage in CDTA was less than optimal for stabilising the artificially-chewed sample and that any compound emulsions that may have formed during chewing were lost during storage.

Separation of the cells in the larger particles was facilitated by CDTA and showed that many of these cells are undamaged (Figure 5) and that the lipid is still present in oleosomes. This was confirmed after staining with Sudan IV (Figure 5c,d) to locate the lipid in undamaged cells, damaged cells and free lipid droplets. Compared with other parenchymatous tissue, the cells of almond seeds are very small and so many cells escape the shearing and crushing forces during chewing, both simulated and in the mouth.

#### 3.4.2. Chewed RA

**Figure 6:**
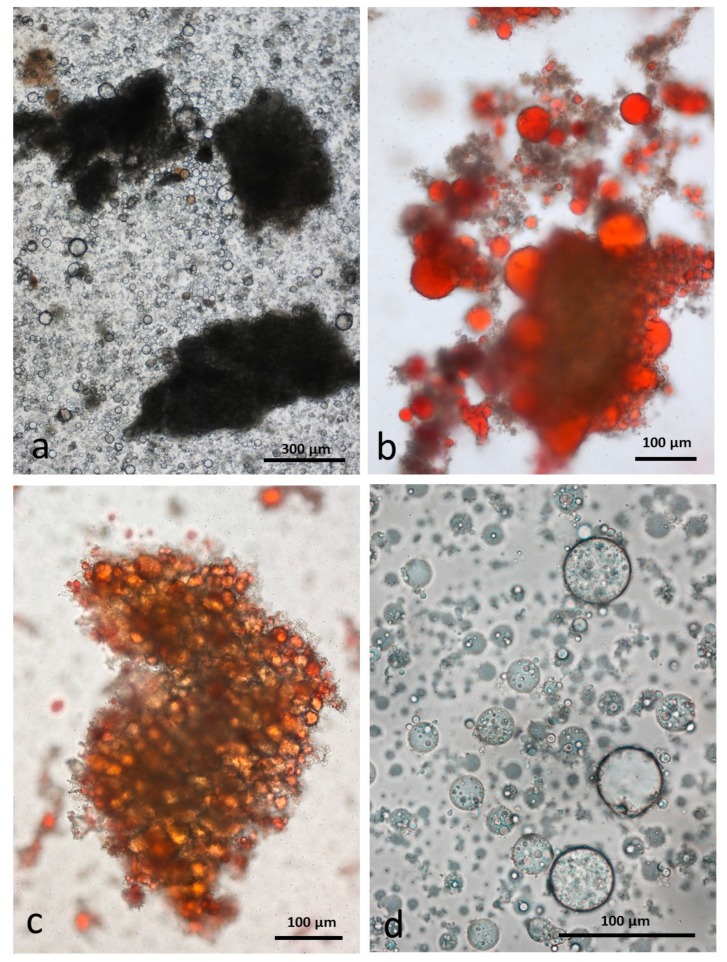
Chewed whole roasted almonds stored in CDTA: (**a**) multicellular particles of almond tissue surrounded by released lipid drops; (**b**) lipid drops stained with Sudan IV adhere to the particles; (**c**) lipid is released from oleosomes during roasting and coalesces within the undamaged cells; (**d**) many of the released lipid drops are in the form of a water-in-oil-in-water emulsion which persist during storage in CDTA.

Roasting is known to make the almond tissue brittle but, as in the raw sample, there are many particles consisting of intact cells in an emulsion of released lipid (Figure 6a). Lipid drops adhere to the surface of these particles (Figure 6b). Although the particles largely contain undamaged cells, the lipid in each cell has coalesced as noted for the unchewed roasted samples (Figure 6c). At higher magnification (Figure 6d) many of the released lipid drops are seen to consist of a water-in-oil-in-water (WOW) compound emulsion. The fact that these compound emulsion drops persisted during storage in CDTA suggests that they are more stable than those in chewed raw almonds. A possible explanation is that during the roasting process when the lipid bodies coalesced, additional interface-stabilising compounds, such as proteins, were released from the cells and these then become active at the surface of the internal water phase drops, effectively preventing them from coalescing. The mechanisms underlying this effect could be the subject of future studies.

#### 3.4.3. Chewed DA

**Figure 7:**
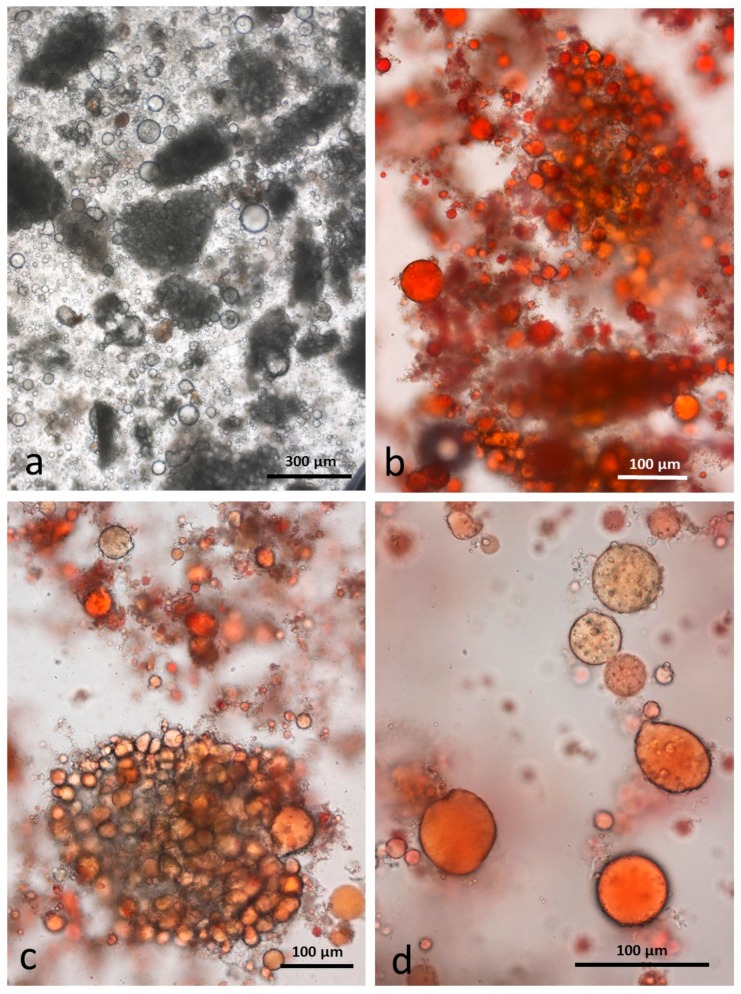
Chewed roasted chopped almonds stored in CDTA: (**a**) multicellular particles of almond tissue surrounded by released lipid drops; (**b**) particles surrounded by released lipid drops; (**c**) undamaged cells are full of coalesced lipid; (**d**) many of the released lipid drops are in the form of a water-in-oil-in-water emulsion which persist during storage in CDTA.

As in the RA, the chewing procedure released numerous particles of undamaged cells surrounded by lipid droplets (Figure 7a). Similarly, each individual uncrushed cell contains coalesced lipid droplets and there are many free lipid droplets released from damaged cells also (Figure 7c,d). These free lipid droplets may consist of a simple single lipid phase, or consist of a stable compound WOW emulsion (Figure 7d).

#### 3.4.4. Chewed AB

**Figure 8:**
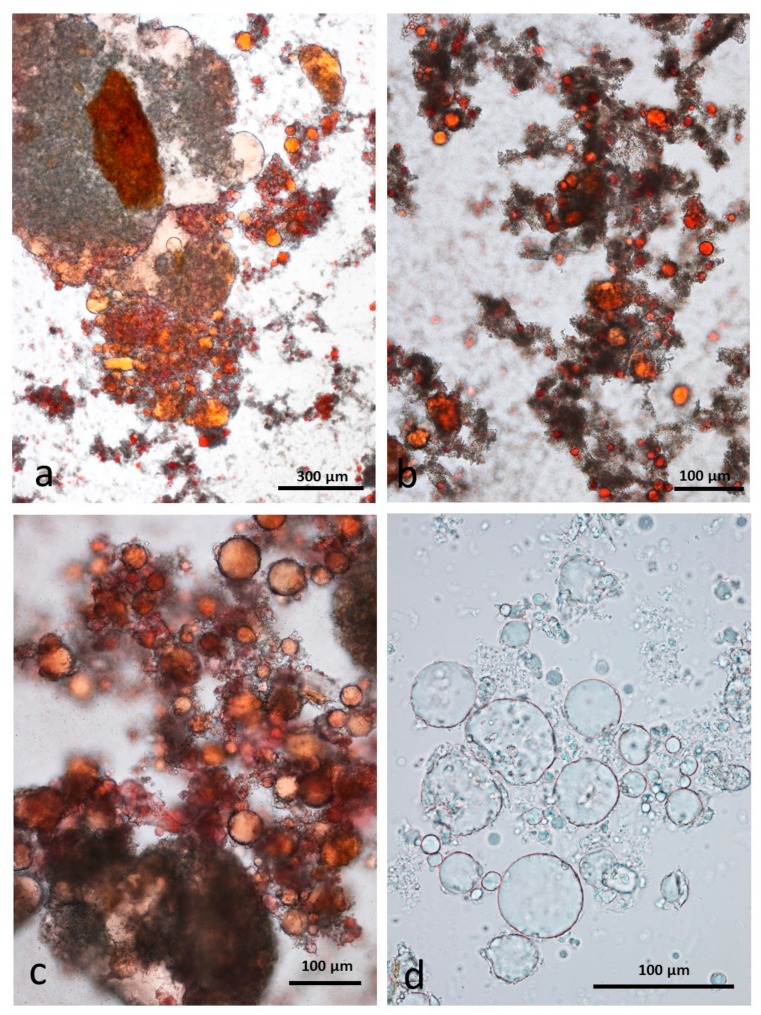
Chewed almond butter stored in CDTA: (**a**) blobs of oily butter containing cell fragments form in the presence of artificial saliva and escape damage from chewing; (**b**) lipid drops and adhering cell fragments released from blobs which have been broken open by chewing; (**c**) lipid drops and protein bodies are closely associated; (**d**) unstained lipid drops appear to have an internal phase but the inclusions are probably protein bodies.

The baseline images of almond butter showed that it consists of a continuous lipid phase in which are suspended small particles of recognisable cells, fragments of cell walls and isolated cell contents. Artificial chewing in the presence of simulated saliva introduced an aqueous phase which resulted in the formation of unchewed blobs of the oily almond butter surrounded by the saliva as in Figure 8a. Where the butter has been subject to chewing, the continuous almond lipid phase formed small lipid droplets adhering to finely divided cell fragments, primarily cell-wall material and protein bodies (Figure 8b,c). At higher magnification (Figure 8d), some lipid drops appear to contain internal water droplets as in the chewed roasted samples, but closer examination showed that they are individual protein bodies which are trapped within the lipid.

### 3.5. Microscopy Examination of Faecal Samples

The samples illustrated come from a representative volunteer.

### 3.6. Control Faeces from Test Diet (No Almonds Consumed) Stained with Sudan IV

**Figure 9:**
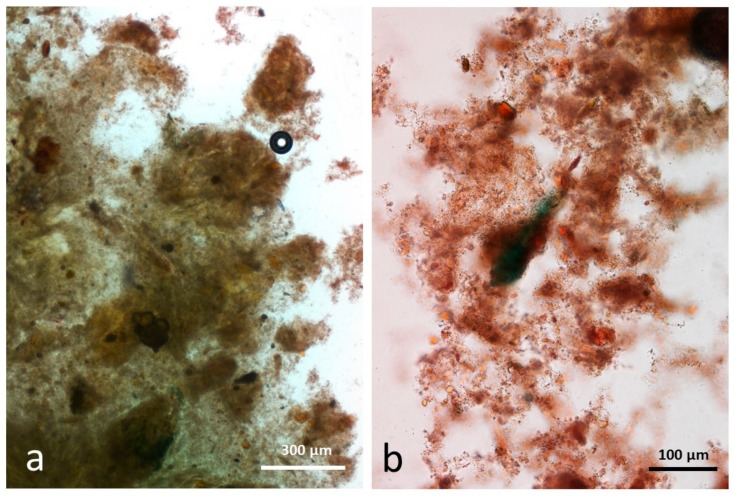
Faecal sample stained with Sudan IV from control diet, no almonds eaten, (**a**,**b**) faeces contain plant remains in a background of micro-organisms and mucin.

Control faecal matter contained recognisable remains of plant tissue in a characteristic background of unstructured material comprising food remains, micro-organisms and mucin (Figure 9a,b). No free lipid drops were observed.

### 3.7. Faeces from Test Diet with NA Stained with Sudan IV

**Figure 10:**
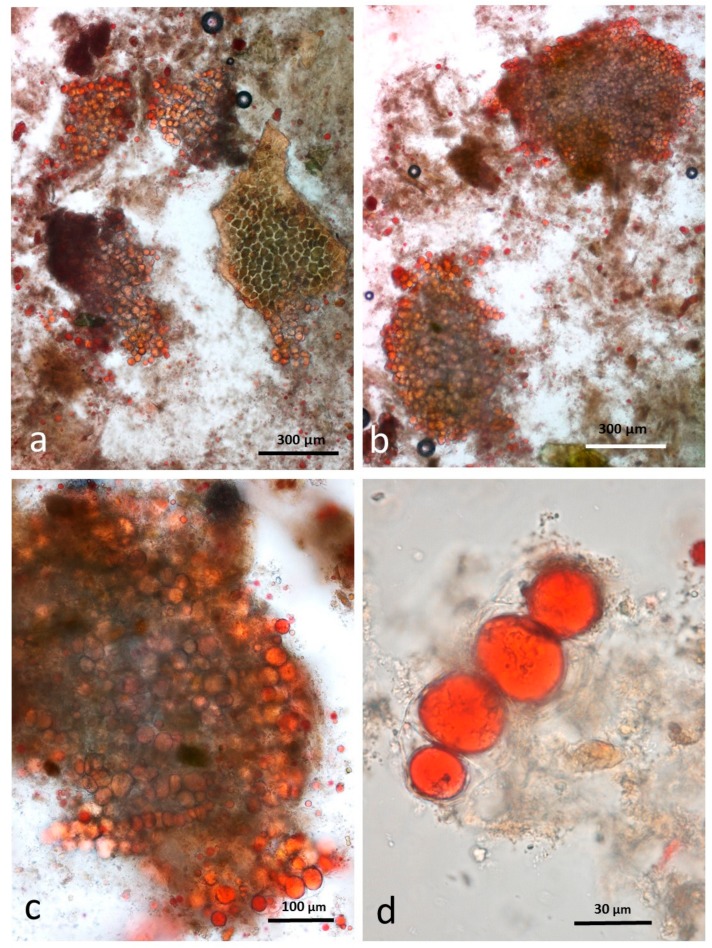
Faecal sample stained with Sudan IV from whole raw almonds diet (**a**) multicellular particles of almond tissue and aleurone cells (right) (**b**) almond particles but few free lipid drops (**c**) lipid in undamaged cells is coalesced (**d**) coalesced lipid confined by cell walls.

This sample contained recognisable multicellular particles of almond tissue in the same size range as that seen in the chewed sample (Figure 10a,b). The right-hand particle in Figure 10a is wheat aleurone tissue with cells similar in size to those of almonds. The aleurone cells also contain lipid but this tissue could be distinguished from almond tissue because it is a single cell layer often still adhering to the brown-pigmented testa. In the faeces, the lipid content of the cells at the periphery of the almond particles are in the form of large drops whereas in the undamaged cells of the chewed raw sample, it is mostly confined to the oleosomes. Without immersion in CDTA to separate the cells, it is not possible to observe whether the lipid in the innermost cells of the larger particles is still within oleosomes as has been seen in almond tissue subjected to in vitro duodenal digestion [15]. There are comparatively few free lipid drops in this sample: close observation showed that lipid drops in Figure 10d are still confined within cell walls.

### 3.8. Faeces from Test Diet with RA Stained with Sudan IV

**Figure 11:**
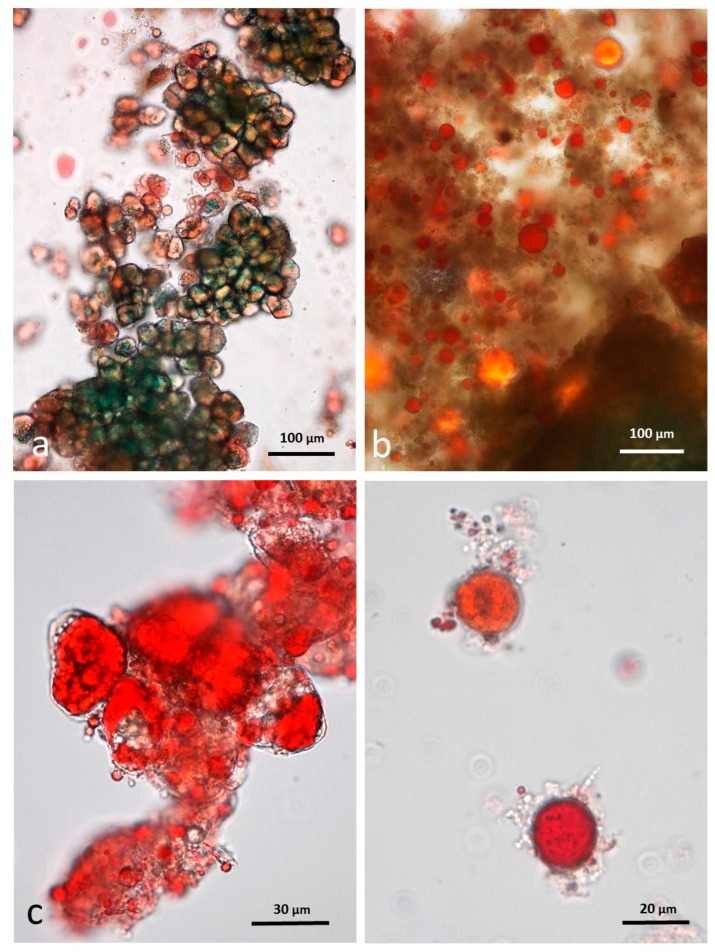
Faecal sample stained with Sudan IV from whole roasted almonds diet: (**a**) multicellular almond particles stained blue/green with marker dye; (**b**) free lipid drops; (**c**) coalesced lipid drops in cells; (**d**) lipid drops with adhering micro-organisms.

Particles of multi-cellular tissue similar in size range to those found in chewed whole roasted almonds survived digestion and are easily recognisable in the faeces (Figure 11a). In this case, the particles are stained blue/green by the marker dye. There are also numerous free lipid drops (Figure 11b) distributed in the faecal matter as well as coalesced lipid still present within cells (Figure 11c). Adhering to the free lipid drops are bacteria from the microbial flora of the intestine (Figure 11d).

### 3.9. Faeces from Test Diet with DA Stained with Sudan IV

**Figure 12:**
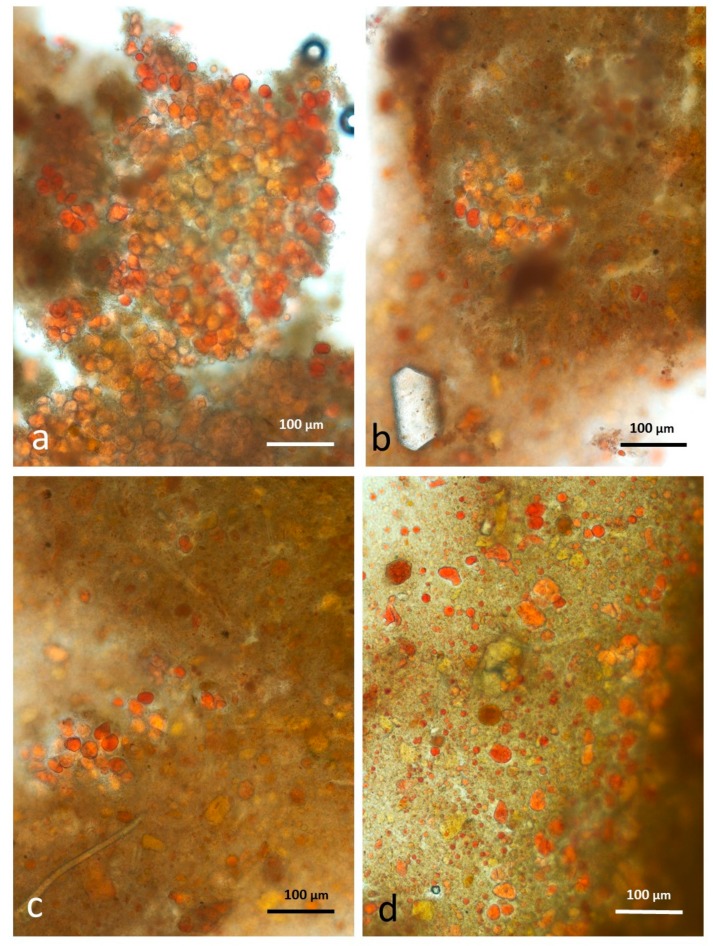
Faecal sample stained with Sudan IV from chopped roasted almonds diet: (**a**,**b**) multicellular almond particles; (**c**,**d**) free lipid drops.

The appearance of chopped roasted almond tissue that had survived passage through the gut is similar to that described for whole roast almonds, with multicellular particles containing coalesced lipid (Figure 12a,b) and an abundance of free lipid (Figure 12c,d).

### 3.10. Faeces from Test Diet with AB Stained with Sudan IV

**Figure 13:**
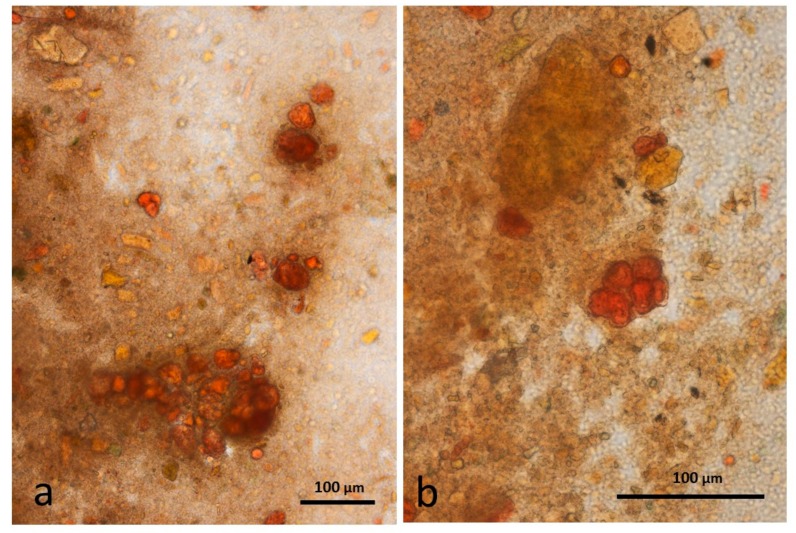
Faecal sample stained with Sudan IV from almond butter diet: (**a**,**b**) small multicellular particles but very few free lipid drops.

Faecal samples from the test diet incorporating almond butter contain smaller multicellular particles of almond tissue than those from whole or chopped almonds (Figure 13a,b), consistent with the fine grinding experienced during production. There are very few free lipid drops in the faeces as the lipid phase liberated during grinding is exposed to lipolytic enzymes during digestion.

## 4. Discussion

This work further validates the findings of loss in metabolisable energy compared to that calculated by the Atwater general factors [10,11]. Further, these results provide insights into the mechanisms by which the almond structure affects the release of lipid and energy. Given the structural diversity of the almond meals analysed in the present study, it is evident that the almond cellular structure plays a crucial role in determining lipid bioaccessibility in the gut. Using in vitro and in vivo techniques, we have recently demonstrated that almond lipid bioaccessibility is significantly affected by the particle size within a food matrix [6]. The importance of nutrient encapsulation within intact cell walls has been previously studied using in vitro models of digestion [5,7]. This present study builds on our previous work [5,6,7,15] by measuring lipid release during simulated mastication from 4 almond meals with different structure and particle size, while simultaneously observing the microstructural changes. It is interesting to note that there was limited lipid digestibility for NA and RA during mastication. In AB, all the intracellular lipids made available by cell-wall rupture, as well as the lipid molecules present at the interface and within the continuous lipid phase are readily available for absorption. The exposure of the remaining intact almond particles to mastication resulted in a further small release of lipid. The coalescence of lipid in RA and DA baseline samples did not limit the digestibility in the mouth, with very similar values of lipid release obtained from the two matrices, slightly above NA. It is worth noting that although roasting did not appear to cause any damage to the cell walls prior to mastication, it could affect the structure of proteins surrounding the coalesced oil bodies typical of roasted samples. If the hydrophilic component of the lipid body stabilizing proteins is reduced or hydrolysed following roasting, the protein could become more lipophilic and thus structurally better suited to stabilising the reversed curvature of a water-in-oil emulsion. However, the stability is likely to be weak and could be compromised through extended storage.

This work further validates our mathematical model of lipid release, showing considerable potential for predicting nutrient bioaccessibility from plant foods which satisfy the model’s assumptions (broadly spherical cells, cell fracture the main mode of failure) [8]. The model was also shown to work without having to combine PSDs from laser diffraction and sieving. This simplifies the experimental work and calculations required and suggests that the model could be used successfully with any method that covers the whole particle size range (see supplementary file).

With the exception of AB, particle size of almonds decreased with mastication and the distributions obtained with NA and RA samples were comparable with our previous investigation [15]. It is known that processing of nuts, such as roasting, chopping and grinding, impacts mastication, particle size and lipid bioaccessibility [7,18,19]. The decrease in size of almond particles, with consequent reduction of intact cell walls, determines the rate and extent of lipid bioaccessibility during digestion. Cassady et al. [20] reported important differences in appetitive and hormone responses after mastication of almonds. Gebauer et al. [11] have recently reported that the number of calories absorbed from almonds in the GIT is strictly dependent on the form in which they are consumed: Atwater factors overestimate the ME of natural raw, roasted and chopped almonds. Overestimation of the ME of nuts could explain data from epidemiological and clinical studies indicating lower body weight of individuals consuming nuts [21,22], who are also less likely to gain weight over time [23,24]. As a result of incomplete macronutrient loss in the upper GIT, it is believed that a large proportion of nutrients from almonds reaches the large bowel, where it is fermented by the microbiota [4,5]. Incomplete rupturing of the cell walls during mastication results in macronutrient encapsulation, which remain inaccessible to digestive enzymes and, if not fermented in the colon, are excreted in faeces. Here, we have further validated the ME results of Novotny et al. [10] and Gebauer et al. [11]. Our microstructural evidence showed recognisable multicellular particles of almond tissue in the same size range as that seen in the chewed sample of the volunteer consuming NA, whereas small multicellular particles of almond tissue were present in the faecal sample of the volunteer consuming AB. Our micrographs provide further evidence of increased energy absorption when cells are broken down during grinding (AB), thus exposing lipid to lipolytic enzymes during digestion. The extensive cell-wall disruption and small particle size of almond butter played a crucial role in the extent of lipid absorption in the gut.

While a relatively small percentage of lipid was released from the almond tissue during mastication, we believe the additional lipid released after mastication [10] is due to a number of mechanisms, including the breakdown of small particles, lipid becoming accessible within intact cells followed by erosion of the particles, or microbial degradation.

In conclusion, we report evidence on the low digestibility of lipids from almonds, which is strictly related to the structure of the meal and the particle size distribution. We believe that food structure influences health through nutrient bioaccessibility in the gut. A greater understanding of the relationship between food structure and nutrient bioaccessibility may be useful to improving bioaccessibility of nutrients that may be poorly available, especially in plant-based diets.

## 5. Conclusions

In conclusion, we report evidence on the low digestibility of lipids from almonds, which is strictly related to the structure of the meal and the particle size distribution. We believe that food structure influences health through nutrient bioaccessibility in the gut. A greater understanding of the relationship between food structure and nutrient bioaccessibility may be useful to improving bioaccessibility of nutrients that may be poorly available, especially in plant-based diets.

## Figures and Tables

**Table 1:** Lipid released during mastication (%) and predicted lipid release (%) from natural raw almonds (NA, *n* = 4), roasted almonds (RA, *n* = 4), diced almonds (DA, *n* = 4) and almond butter (AB, *n* = 4) due to sample processing and mastication. Values represent the average ± SD (standard deviation).

Almond Meal	Lipid Released Due to Mastication (%)	Predicted Lipid Release (%) *	Total Lipid Potentially Available for Digestion (%) **
NA	8.9 ± 0.7	9.6	8.9 ± 0.7 ^b^
RA	11.8 ± 1.1 ^a^	12.6	11.8 ± 1.1 ^b^
DA	12.4 ± 0.8 ^a^	9.6	12.4 ± 0.8 ^b^
AB	6.2 ± 0.4	6.4	94.0 ± 4.6

* Sieving, average of *n* = 2; ** Referred to % of total lipid; ^a^ *p* < 0.001 vs. AB; ^b^ *p* < 0.001 vs. AB.

## Supplementary Materials

The following are available online at http://www.mdpi.com/2072-6643/10/2/213/s1.

## Abbreviations

NA	Natural Almonds
RA	Roasted Almonds
DA	Diced Almonds
AB	Almond Butter
GIT	Gastrointestinal Tract
ME	Metabolisable Energy
HAS	Human Salivary Amylase
PSD	Particle Size Distribution
